# Pathogenesis of Extraarticular Manifestations in Rheumatoid Arthritis—A Comprehensive Review

**DOI:** 10.3390/biomedicines11051262

**Published:** 2023-04-24

**Authors:** Joško Mitrović, Stela Hrkač, Josip Tečer, Majda Golob, Anja Ljilja Posavec, Helena Kolar Mitrović, Lovorka Grgurević

**Affiliations:** 1 Division of Clinical Immunology, Rheumatology and Allergology, Department of Internal Medicine, Dubrava University Hospital, School of Medicine and Faculty of Pharmacy and Biochemistry, University of Zagreb, Avenija Gojka Šuška 6, 10000 Zagreb, Croatia; 2 Polyclinic for the Respiratory Tract Diseases, Prilaz Baruna Filipovića 11, 10000 Zagreb, Croatia; 3 Department of Rheumatology and Rehabilitation, Zagreb University Hospital Center, University of Zagreb School of Medicine, Kišpatićeva 12, 10000 Zagreb, Croatia; 4 Center for Translational and Clinical Research, Department of Proteomics, School of Medicine, University of Zagreb, 10000 Zagreb, Croatia; 5 Department of Anatomy, “Drago Perovic”, School of Medicine, University of Zagreb, 10000 Zagreb, Croatia

**Keywords:** rheumatoid arthritis, pathogenesis, inflammation mediators

## Abstract

Rheumatoid arthritis (RA) is among the most prevalent and debilitating autoimmune inflammatory chronic diseases. Although it is primarily characterized by destructive peripheral arthritis, it is a systemic disease, and RA-related extraarticular manifestations (EAMs) can affect almost every organ, exhibit a multitude of clinical presentations, and can even be asymptomatic. Importantly, EAMs largely contribute to the quality of life and mortality of RA patients, particularly substantially increased risk of cardiovascular disease (CVD) which is the leading cause of death in RA patients. In spite of known risk factors related to EAM development, a more in-depth understanding of its pathophysiology is lacking. Improved knowledge of EAMs and their comparison to the pathogenesis of arthritis in RA could lead to a better understanding of RA inflammation overall and its initial phases. Taking into account that RA is a disorder that has many faces and that each person experiences it and responds to treatments differently, gaining a better understanding of the connections between the joint and extra-joint manifestations could help to create new treatments and improve the overall approach to the patient.

## 1. Introduction

Rheumatoid arthritis (RA) is among the most prevalent and debilitating autoimmune inflammatory chronic diseases, which affects primarily the synovial membrane of joints and is characterized by a symmetrical destructive polyarthritis as well as affecting extraarticular tissue. The presence of certain autoantibodies is a hallmark of the disease, and they include rheumatoid factor (RF), an antibody recognizing the Fc-tail of immunoglobulin G (highly sensitive but less specific to RA), and specific antibodies against proteins modified by citrullination (antibodies to citrullinated protein antigens—ACPAs) and carbamylation (anti-CarP antibodies) [1,2].

The presence of RF in RA patients may predispose them to more erosive joint disease and make them more likely to develop extraarticular manifestations (EAMs), especially rheumatoid nodulosis, vasculitis, Felty syndrome (FS), and serositis [3,4,5]. Even though the hallmark feature of RA is the involvement of peripheral joints, the clinical presentations are variable. Due to the systemic nature of the disease, RA is associated with increased acute-phase response and can lead to a number of extraarticular manifestations (EAMs), which are an important, albeit partially poorly understood, aspect of this disease [1].

Studies show that EAMs are relatively common (affecting around 40% of RA patients) and could be attributed to a large number of factors and their mutual interactions [6,7,8]. However, there are no strict criteria for the definition of EAMs, and diagnosis is reliant on clinical judgment [9]. Given the backdrop of RA, there could be a link between certain clinical characteristics, laboratory parameters, medications, and EAMs [10]. Among subject groups with RA, different factors, such as RF and antinuclear antibody (ANA) positivity, joint erosions, methotrexate, and systemic glucocorticoid use were significantly associated with EAMs [6,11]. However, lifestyle factors, such as smoking, and hereditary traits, such as male sex and certain HLA gene homozygosity (certain DRB1*04 subtypes), were also shown to be significantly associated with EAMs [8]. Chronic systemic inflammation can be the result of complex interactions between genetic and environmental factors, which lead to certain innate or adaptive immune responses. Identification and adequate treatment of these manifestations are of great importance as they have been linked with increased morbidity and mortality risk [6,12]. RA can involve all organ systems, with a wide range of symptoms. These can include a multitude of manifestations such as skin and ocular manifestations, cardiovascular disease, pleuropulmonary manifestations, Felty syndrome, hematologic abnormalities, rheumatoid vasculitis, neurological, manifestations, amyloidosis, and hepatic and renal involvement [13]. Several EAMs also present with different timeframes of occurrences in the disease—e.g., interstitial lung disease may even appear before the onset of classical RA symptoms, or in longstanding disease, while vasculitis, FS, or meningitis often occur later in the disease course [5,14,15,16]. Although these numerous disease manifestations are well described throughout the literature, and physicians are aware of their importance, the data collected is inconsistent and the cause of a lot of EAMs remains unclear [9]. In order to gain more precise definitions for RA EAMs, improve our understanding of them, and facilitate early recognition, but also to ensure more adjusted treatment for patients, a better insight into the pathophysiological aspects of these manifestations is needed. The pathophysiology of several more common EAMs affecting different organ systems with significant morbidity and mortality are discussed in this review.

## 2. Cardiovascular Disease

There is a significant amount of evidence indicating greater rates of coronary artery disease, clinical heart failure, and stroke events among individuals with RA. Atherosclerosis, usually mentioned as a complication of RA, could be handled as an extracellular manifestation that can lead to accelerated cardiovascular events [17]. Cardiovascular disease (CVD) is the leading cause of death in RA patients [18,19]. Meta-analyses have shown that patients with RA have a 48% increased risk of CVD incidents compared to the general population while CVD mortality is increased by around 52% [20,21]. Although traditional risk factors have an important contribution to CVD risk, this increased risk cannot be completely explained by them [22]. Therefore, CVD could be discussed not only as a comorbidity but also as an EAM of RA. Regarding their influence on mortality and morbidity in RA patients, but also growing evidence of pathogenic relation to RA, atherosclerosis and non-ischemic heart disease will be discussed in this section.

### 2.1. Atherosclerosis

Today it is known that inflammation, in addition to lipid accumulation, has an important role in atherosclerosis pathogenesis, and therefore also CVD [23]. Failure to resolve inflammation in atherosclerotic plaques caused by disbalance between the specialized pro-resolving mediators (SPMs) and the proinflammatory lipids could be one of the important mechanisms of atherosclerosis progression, but also a new treatment target [24]. 

#### 2.1.1. Endothelial Dysfunction

Endothelial dysfunction is the first step in atherogenesis and its development in RA is associated with multiple factors: systemic inflammation, proinflammatory cytokines (TNF-α, IL-6, IL-1, IFN-γ, and IL-17), oxidative stress, disbalance of certain hormones (leptin, resistin, and adiponectin), increased expression of adhesion molecules, altered endothelial progenitor cell function, immune dysregulation, genetic predisposition, as well as traditional factors such as diabetes mellitus, arterial hypertension, and high body mass index [25]. Systemic inflammation and immune dysregulation seem to impair production of nitric oxide (NO), a molecule which has an important protective role in arteries [26]. TNF-α, an important cytokine in RA pathogenesis, was shown to inhibit eNOS (endothelial nitric oxide synthase) promoter activity and destabilize its mRNA leading to lower NO levels in endothelium [27,28]. Another mechanism of lower nitric oxide synthase (NOS) activity in RA seems to be higher levels of asymmetric dimethylarginine (ADMA) which competitively inhibits this enzyme and is associated with higher CVD risk in RA patients [29,30,31]. There are several proposed mechanisms for higher levels of ADMA in RA: inhibition of its degradation enzymes, dimethylarginine dimethylaminohydrolase (DDAH), by TNF-α, reduced DDAH enzyme in the hypoxic environment of inflamed synovia leading to higher ADMA plasma levels, and enhanced production of ADMA due to enhanced protein arginine methyltransferase (PRMT) enzyme gene expression in endothelial cells caused by higher oxidized LDL (oxLDL) levels in RA patients [31]. Furthermore, Akhmedov et al. in their recent study showed that TNF-α caused time-dependent endothelial dysfunction in RA murine models and increased levels of LOX-1 (scavenger LDL receptor on endothelial cells) [32]. The downstream activity of this receptor increased arginase 2 activity via NFkB which led to eNOS uncoupling—a process in which this enzyme produces increased superoxide anions but decreased NO levels [32,33,34].

Another mechanism of endothelial dysfunction in RA patients could be explained by microparticles (MPs) and microparticles that form immunocomplexes (MP-ICs). These extracellular vesicles, found in a physiologic state carrying proteins, nucleic acids, receptors, and other macromolecules between different cells, found to be increased in RA patients are shown by Atehortúa et al. to increase endothelial expression of the adhesion molecules CD54 and CD102, and the production of inflammatory mediators, such as IL-6, CCL2, and CCL5 in a dose-dependent way. These particles also increased monocyte adhesion in the macrovasculature (but not in the microvasculature) and decreased cell adhesion, depolymerized actin filaments, and triggered cell death, which could all lead to increased permeability of endothelium [35]. TNF-α was also shown to increase cell adhesion molecule (CAM) expression through stimulation of NF-κB and p38MAPK and by the abovementioned NO reduction since NO reduces CAM expression [28,36,37,38].

#### 2.1.2. Lipoprotein Abnormalities

Low-density lipoprotein cholesterol (LDL-C) of the vessel intima and its oxidation is an important step of atherosclerosis joined with endothelial dysfunction [26]. Increased LDL-C enhances this process and dyslipidemia is a well-known traditional risk factor for CVD, as well as diseases related to dysregulated lipid metabolism such as diabetes mellitus and metabolic syndrome [39,40]. Increased prevalence of the latter was observed in RA patients, as well as an increased prevalence of insulin resistance which is linked to endothelial dysfunction and lipid metabolism dysregulation [41]. On the other hand, an interesting phenomenon called “lipid paradox” is described in RA patients: patients with lower LDL-C levels have higher CVD risk than patients with moderate LDL-C levels [42,43]. Inflammatory processes could be attributed to this paradox, although it is still not fully understood [42]. Part of the explanation might also be that the L5 fraction (the most electronegative subfraction of LDL-C) could be responsible for higher CV risk in RA patients. Chang et al. showed significantly higher levels of this fraction in RA patients compared to healthy controls [44]. L5 LDL-C levels were also higher in RA patients with subclinical atherosclerosis and were positively correlated to the extent of carotid artery atherosclerosis and disease activity. Furthermore, a higher L5 percentage in RA patients was associated with significantly higher expression of the gene encoding integrin CD11c which is linked to plaque formation [45]. L5 fraction also led to foam cell formation and increased the expression of IL-6, IL-8, and TNF-α in in vitro assays as well as the expression of the LOX-1 receptor [44,45]. Higher expression of LOX-1 was linked to increased vascular tissue levels of oxLDL in both mice and RA patients, in spite of normal lipoprotein levels [32]. Another explanation for the “lipid paradox” could be abnormal high-density lipoprotein (HDL) function and structure which is known for its protective role in atherosclerosis [46]. Under inflammatory conditions, the proteome and lipidome of HDL may change, leading to the development of pro-inflammatory HDL (piHDL). Pro-inflammatory HDL is different in structure than normal HDL: it lacks apolipoprotein A-I (apoA-I) and paraoxonase 1 (PON1) but is enriched with oxidized phospholipids and lysophospholipids, free cholesterol, free fatty acids (FFAs), and triacylglycerols (TAGs). Additionally, piHDL contains pro-inflammatory proteins such as serum amyloid A (SAA) and ceruloplasmin. As for its function, although it possesses the ability to enhance the inflammatory response to infections, piHDL can also promote the oxidation of low-density lipoprotein (LDL), cause endothelial dysfunction, stimulate the activity of monocytes, enhance the secretion of pro-inflammatory molecules, and is less effective in cholesterol efflux activity [47]. Accordingly, levels of piHDL correlated with disease activity in RA patients [48]. Furthermore, RA disease activity was shown to be associated with lower HDL antioxidative function and decreased cholesterol efflux activity in in vitro macrophages [49,50]. Both of these impaired HDL functions are linked to higher myeloperoxidase enzyme (MPO) activity [49]. This enzyme has been demonstrated to carry out nitration and chlorination of apolipoprotein A-I (apo A-I) in HDL and bind to it, thus inducing oxidation. All of these processes were found to be associated with the inhibition of cholesterol efflux from macrophages [51]. Some studies have demonstrated that antibodies against HDL and its components were not only present at RA onset but were also detectable prior to the establishment of a clinical diagnosis. These antibodies did not correlate with traditional CV risk factors but were related to biological processes involved in atherosclerosis [52].

#### 2.1.3. Role of Immune Cells

**Figure 1:**
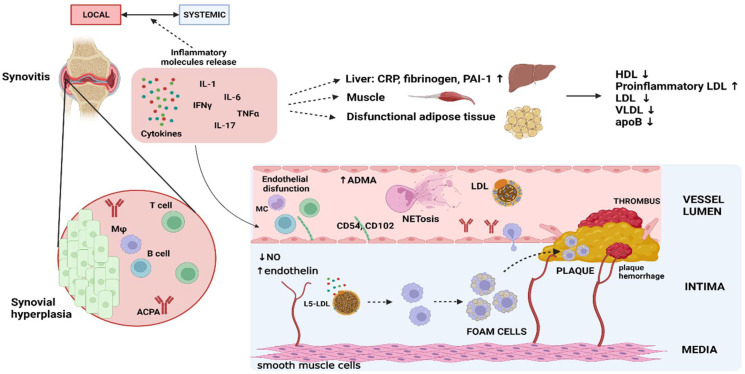
Depiction of discussed pathophysiological mechanisms in RA leading to atherosclerosis. MC/Mφ = monocyte/macrophage. Created with BioRender.com.

Monocyte recruitment following endothelial dysfunction is an important step in atheroma formation [26]. However, not only monocytes/macrophages but also neutrophils, T lymphocytes, and B lymphocytes all seem to have their role in atherosclerosis [23]. Monocytes migrating to atherosclerotic plaques, differentiating to macrophages which form foam cells by taking up LDLs, is a well-known part of atherosclerosis [26]. Studies show that an increased proportion of CD14 + CD16+ monocytes is associated with more severe coronary artery disease (CAD) [53]. This subtype of monocytes is found to be elevated in RA patients and linked to a higher risk of subclinical artery atherosclerosis [53,54]. A study by Dragoljevic et al. showed that cholesterol efflux alterations in hematopoietic stem and progenitor cells (HSPCs), found in RA murine models and RA patients, could be a possible link between inflammation in RA and the aforementioned elevated monocyte levels. These efflux alterations led to HSPC proliferation which resulted in elevated levels of monocytes, neutrophils, and thrombocytes. This process seems to be driven by RA-associated cytokines (GM-CSF, TNF-a, IL-1 β, and IL-6). Cholesterol efflux alterations remained in mature myeloid cells and are proposed to be associated with impaired lesion regression [55]. Furthermore, the procoagulant activity of monocytes was observed to be enhanced by IL-6 (a cytokine known to be elevated in the sera of RA patients), probably through tissue factor (TF) induction, leading to increased risk of thrombotic events [56,57]. Monocyte recruitment to plaques was also found to be linked with the IL-6 family. A lack of gp-130 (a signal transducer used by the IL-6 family) in mice hepatocyte cells led to lower macrophage levels in plaques and lower CCL2 expression. One mechanism for this could be lower serum amyloid A (SAA) levels, which were shown to increase CCL2 expression and macrophage migration. Furthermore, certain polymorphisms of the human gp-130 gene (IL6ST) were found to be associated with CAD, especially with ostial vessel plaques [58]. In patients with unstable angina, a subset of T lymphocytes (CD4 + CD28null T-cells) that is often associated with older age and infections, can be found, unlike in those with stable disease and RA patients with extraarticular involvement [59,60,61,62,63]. RA patients with an expansion of these cells were shown to have impaired flow-mediated vasodilatation and increased intima–media thickness compared to RA patients without CD4 + CD28null T-cells [64]. Neutrophils have been found in both murine and human atherosclerotic lesions and studies suggest neutrophils could contribute to endothelial dysfunction, monocyte recruitment and activation, foam cell formation, plaque destabilization, and weakening of the fibrous cap [65]. The release of neutrophil extracellular traps (NETs), as one of the antimicrobial neutrophil activities, was found to be increased in RA patients compared to healthy controls, and their release positively correlated with disease activity and oxidative and inflammatory plasma markers. NETosis products—cell-free nucleosomes were found to be a specific marker for identifying RA patients with early atherosclerosis [66]. Furthermore, in vitro tests showed that treatment of neutrophils with the serum of RA patients led to increased NETosis compared to healthy controls and that the supernatants of those cultures increased expression of genes related to adhesion molecules, pro-thrombotic, and proinflammatory mediators in endothelial cells (HUVEC) and peripheral blood mononuclear cells (PBMCs). The addition of tocilizumab, infliximab, or DNase to patients’ serum in this process led to reduced expression of these molecules, which suggests a role of RA patients’ serum proinflammatory cytokines TNF-α and IL-6 in NET-mediated inflammation and atherothrombotic processes [66,67]. The discussed pathophysiological mechanisms involved in atherosclerosis are depicted in Figure 1.

### 2.2. Non-Ischemic Heart Disease

Patients with RA are at increased risk of heart failure, which cannot be fully explained by the increased risk of ischemic heart disease. Although higher disease activity was associated with all types of heart failure, the connection is most pronounced in non-ischemic heart failure [68]. Regarding this, mechanisms of atherosclerosis development described in the previous subsection cannot completely explain the higher risk for heart disease in RA patients. However, data on the pathogenesis of non-ischemic heart disease is scarce. Radiological imaging, including magnetic resonance imaging and positron emission tomography/computed tomography, shows that disease activity was linked to increased myocardial inflammation and fibrosis, which suggests that systemic inflammation, a hallmark of RA, could lead to stiffening of ventricles and therefore systolic and diastolic dysfunction [69,70,71]. Several cytokines known to have an important role in RA are also linked to non-ischemic heart disease.

Continuous exposure to TNF-α in rodent models led to left ventricle dysfunction, remodeling, and dilatation, and disrupted collagen structure [72]. Although the mechanisms of this effect are not understood, those suggested include the induction of certain matrix metalloproteinases, activation of the sphingomyelinase pathway, and inhibition of β-adrenergic responsiveness [72,73,74]. IL-1 was also shown to depress myocardial activity and anti-IL1 therapy showed improvement in left ventricular function in RA patients [75]. There are several proposed mechanisms for this: inhibition of β-adrenergic responsiveness by inducing the uncoupling of the β-adrenergic receptor (β-AR) from the adenylyl cyclase (AC) and from the calcium (Ca^2+^) channels; inhibition of phospholamban (an important regulator of cardiac contractility); and increasing production of ceramide and NO, both leading to the abovementioned uncoupling of Ca^2+^ channels from β-AR [76]. The presence of higher concentrations of proinflammatory interleukins in RA patients may result in increased attachment to their receptors and potentially lead to myocardial dysfunction due to the mechanisms mentioned above, although this does not directly suggest a cause [77]. Furthermore, higher myocardial interstitial citrullination staining was observed in RA patients, and elevated levels of ACPAs were associated with a higher left ventricular mass index, suggesting that these antibodies, as a hallmark of RA, could also play a role in myocardial dysfunction [77,78,79]. Although the underlying mechanism is yet to be discovered, it could be assumed that ACPAs form immunocomplexes and lead to local inflammation similar to processes in rheumatoid synovia, which then causes myocardium remodeling [77,80]. 

## 3. Pulmonary Manifestations

Pulmonary manifestations of RA are one of its most common EAMs, with reports of occurrence ranging from 10% to 67% [81,82]. Pulmonary manifestations usually occur after joint manifestations, but lung disease may also predate classic symptoms of RA, including arthritis, by many years [83]. Symptomatic large or small airway disease manifesting with cough or shortness of breath has been reported in 30% of patients with RA [84,85,86]. Pulmonary manifestations can manifest in different forms, affecting different lung compartments: in the form of interstitial lung disease (ILD), pulmonary nodules, and pneumoconiosis, affecting lung parenchyma, as well as pleural effusions, affecting the pleura (discussed later in the section “Membrane involvement”), or obliterative/constrictive bronchiolitis and bronchiectasis, affecting the lower airways [13]. Large airways can also be affected, resulting in cricoarytenoiditis as well as vasculature, which manifests as vasculitis and pulmonary hypertension [86]. Nodules of the lung parenchyma may appear in the form of rheumatoid nodules (discussed below in a separate section) or due to pneumoconiosis, an inflammatory lung condition that occurs due to extensive occupational exposure to silica, asbestos, and coal [13]. Pneumoconiosis associated with RA, also known as Caplan syndrome, is a rare clinical condition characterized by the development of multiple nodules, mainly in the lung periphery of affected patients [13,86]. Reports vary on the respective prevalence of different pulmonary manifestations. However, ILD and rheumatoid nodules are reported to be the most common parenchymal pulmonary manifestations, while bronchiectasis may be the most common lower airway manifestation [86]. The pathogenesis of these more common pulmonary manifestations of RA will be discussed below. 

### 3.1. Interstitial Lung Disease

ILD is the most common of RA pulmonary manifestations, as signs of it have been reported in 30–60% of RA patients [85,86]. However, ILD is commonly asymptomatic, as studies showed that among these subjects, 76% of patients with RA-ILD (or 90% of all RA patients) had no clinical signs or clear symptoms of ILD [86,87]. Others report that symptomatic ILD occurs in 5–17% of patients with RA [83,88,89]. ILD is one of the most severe EAMs of RA (next to cardiovascular disease), as the average patient survival equals 3 years [1].

**Figure 2:**
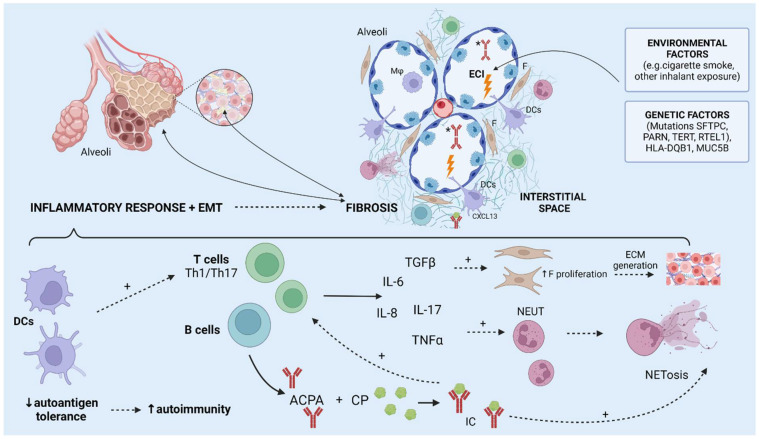
Illustration showing the described pathophysiological mechanisms leading to the development of interstitial lung disease. EMT = epithelial-to-mesenchymal transition, ECI = epithelial cell injury, DCs = dendritic cells, CP = citrullinated proteins, F = fibroblast, CP = citrullinated proteins, * IgA ACPAs. Created with BioRender.com.

ILD in RA predominantly manifests in the patterns of usual interstitial pneumonia (UIP), and nonspecific interstitial pneumonia (NSIP) in addition to other, less frequent patterns such as organizing pneumonia, lymphoid interstitial pneumonia, and diffuse alveolar damage [14]. The exact pathophysiological mechanisms of ILD remain poorly understood. There are multiple factors thought to be involved in the pathogenesis of RA-related ILD. Two possible pathways for RA and ILD coexistence have been suggested: the first starts with joint inflammation which spreads to the lungs and causes an inflammatory response, including a Th1-, Th2-, and Th17-specific cytokine cascade, which subsequently causes the progression of inflammation to fibrosis and clinically presents as a non-UIP pattern [90]. The second proposed pathway of ILD development is by the aberrant response to alveolar microinjuries conditioned by genetic and epigenetic factors which leads to the activation of myofibroblasts and epithelial cells, which in turn results in progressive fibrosis in which inflammation is not dominant, leading to a UIP pattern similar to idiopathic pulmonary fibrosis (IPF) [90]. There is even evidence that supports the theory of the mucosal origin of RA-related autoimmunity, which hypothesizes that mucosal tissue might be the origin of initiating events that precede systemic autoimmunity resulting in symptomatic RA [91]. Namely, IgA ACPAs have been found in sputum samples of individuals without manifest RA but with risk factors for RA, and higher levels of ACPAs have been found in bronchoalveolar lavage fluids than in the serum of individuals with early RA without lung manifestations [92,93]. Both genetic and environmental factors, such as smoking, are thought to play a role in the pathogenesis of RA-ILD [87]. Several HLA genes have been linked to a predisposition to RA-related lung diseases, such as HLA-DQB1*03:01, HLA-DRB1*15, and HLA-DRB1*16, as well as specific mutations in other genes, such as SFTPC, RTEL1, and TERT [94,95]. However, epithelial cell injury (primarily caused by smoking) of airways and alveoli is thought to be the first process leading to the pathogenesis of RA-associated lung disease [14]. In genetically predisposed individuals, repeated damage of airway epithelial cells, causing antigenic stimulation, can result in lung dendritic cell (DC)-mediated development of autoimmunity by causing the abrogation of autoantigen tolerance, which in turn causes autoantibody production and T-cell activation [14,96]. Dendritic cells (DCs) are crucial to this process as they regulate T-cell activation in the lungs by expressing modulatory ligands and chemokine receptors [96]. Furthermore, the pathophysiologic role of ACPAs in RA and RA-associated lung disease has not been fully elucidated, although higher serum ACPA levels have been found in affected individuals, and higher levels have been shown to correlate to airway disease and ILD [14]. ACPAs can, however, contribute to cell injury, by forming immune complexes and causing the release of pro-inflammatory cytokines such as IL-6, IL-8, and TNF, and can also cause a release of NETs [97,98]. Additional mechanisms which contribute to RA-ILD include Th17-cell-mediated immunity involving cytokines IL-17A and TGFß1, which lead to the proliferation of fibroblast and extracellular matrix generation and subsequently to fibrosis [99,100]. Furthermore, smoking is an important factor in the pathogenesis of RA-related lung disease, as several components of cigarette smoke have been linked to RA pathogenesis. Polycyclic aromatic hydrocarbons (PAHs) seem to be particularly interesting, as they have been shown to activate a particular transcription factor (aryl hydrocarbon receptor, AHR) in synovial DCs, which causes increased production of IL-6 [101]. Nicotine also seems to have an important role in ILD pathogenesis, as it has been shown to induce the release of NETs, which are a source of ACPAs and promote cytokine activation and release by synoviocytes [102,103]. Additionally, smokers have been shown to have increased numbers of DCs in the interstitial and airway tissue [104]. Links have also been found to other potential causes of enhanced autoimmunity, such as disturbance of the microbiota, known as dysbiosis [14]. Moreover, gastroesophageal reflux disease might influence dysbiosis and mediate the introduction of culprit pathogens into the airway, in addition to its previously described potential role in the progression of fibrotic ILD [105]. There is evidence suggesting differences between microbiota species in individuals with or without RA, which might modulate the inflammatory response, but these mechanisms are still poorly understood [14]. Taken together, these intrinsic and extrinsic factors lead to an inflammatory response, which together with epithelial-to-mesenchymal transition causes fibrosis and remodeling of the interstitium, finally resulting in ILD (Figure 2) [106,107]. 

### 3.2. Bronchiectasis

Bronchiectasis in RA is considered to be a cause of lower airway disease, which has been found by radiographic imaging and/or pulmonary function tests in as many as 30% of RA patients, and “bronchial dilatation” has been described in up to 41.3% [82,86]. However, although they can be detected in a greater number of patients, they are not necessarily accompanied by clinical symptoms [86]. Changes caused to the lung parenchyma by ILD can also affect other lung components, thereby possibly even causing bronchiectasis, e.g., by causing traction which can lead to bronchiectasis development [108]. Bronchiectasis may also develop independently, without RA-associated ILD. The pathogenesis of RA-related bronchiectasis is still poorly understood [108]. The proposed mechanisms of bronchiectasis development include airway damage caused by autoimmunity and possibly also chronic infections, which might be enhanced in the setting of RA and concomitant therapy, particularly biological agents [86,109]. The main changes which cause the development of bronchiectasis are impaired mucociliary clearance, chronic infection, persistent inflammatory response, and abnormal airway remodeling [108]. A proposed pathophysiological model states that extensive pulmonary inflammation caused by infection may lead to damage of the respiratory epithelium, hindered mucociliary apparatus, and subsequent bacterial overgrowth [110]. Resulting infections perpetuate inflammation, followed by cytokine and proteinase production which further damage the respiratory epithelium and therefore cause a vicious cycle of infection, inflammation, and airway damage [110]. Additionally, it has even been hypothesized that bronchiectasis might predate RA and contribute to its development by causing prolonged antigenic stimulation through chronic infection [111]. The recurring inflammation causes higher levels of IL-17a, IL-1β, and IL-8, which have been reported in bronchiectasis patients with exacerbations and are therefore implicated in the pathogenesis [112,113]. Furthermore, patients with bronchiectasis, even without RA, have been shown to have increased serum ACPAs compared to healthy controls, suggesting that recurring infections alone might be sufficient for ACPA generation, while ACPA positivity has been connected to a significant difference in high-resolution-computed-tomography-confirmed bronchiectasis and airway thickening [114,115]. The associations and underlying pathogenesis of these conditions still require further research [111]. 

## 4. Rheumatoid Nodules

Rheumatoid nodules, as a form of cutaneous disease manifestation, represent the most common EAMs in RA [116]. They usually appear on extensor surfaces or skin subjected to repeated pressure and/or trauma [117]. Rheumatoid nodules may also appear in locations other than the skin, most commonly in the lung [117]. These parenchymal pulmonary nodules (pulmonary rheumatoid nodules) have a reported prevalence of <0.4–32% in RA patients and are considered to be mostly asymptomatic [118,119] The exact pathogenesis of pulmonary rheumatoid nodules has not been completely elucidated, although they are considered to correspond to rheumatoid nodules appearing in the subcutaneous tissue due to their similar pathohistological appearance. Histologically, rheumatoid nodules correspond to immune-mediated granulomas with central necrosis, surrounded by HLADR^+^ (CD64^+^ and C4d^+^) macrophages, lymphocytes, and histocytes [117,120,121]. However, B-cell infiltrates have been described in pulmonary nodules, while they have not been found in subcutaneous nodules [122]. Common mechanisms thought to be involved in the pathogenesis are local injury, small capillary vasculitis, and effects of immune complexes and proteolytic enzymes [120,123]. Acute vasculitis may also rarely be seen in the vessels surrounding the nodule, or even a necrotic blood vessel at the center [116]. Trauma to small blood vessels, more precisely endothelial damage, is believed to play a role in nodule formation, leading to the aggregation of RF complexes and activation of monocytes and macrophages, which secrete IL-1, prostaglandin E2, TNFα, and TGFβ, as well as proteases, collagenases, and chemotactic factors [116,117,120]. This is followed by fibrin deposition and cytolytic and enzymatic degradation which causes necrosis. This process is contained by palisading macrophages [117]. Additionally, similar to other forms of rheumatoid nodules, higher levels of IL-1, together with IL-12, IL-18, IL-15, and IL-10, have been reported in pulmonary rheumatoid nodules. These findings indicate that the same cytokine profile as in RA synovial lesions, which is attributed to a Th1-driven inflammatory process, is present in rheumatoid nodules. This gives further credence to the idea that these nodules may be Th1-mediated granulomas [124]. Interestingly, ectopic lymphoid follicles, which are considered characteristic of rheumatoid synovial lesions, were found in samples of two pulmonary nodules [122]. This further underlines the need for further investigation of the similarity of underlying mechanisms.

## 5. Membrane Involvement

**Figure 3:**
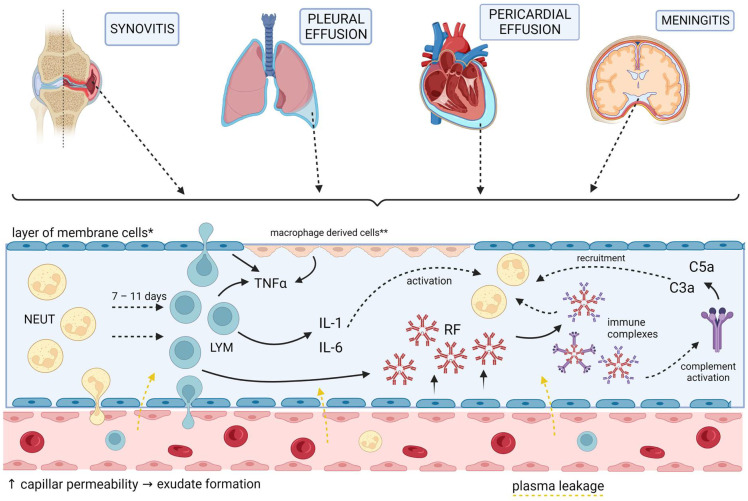
Illustration showing proposed common mechanisms of local inflammation in RA-related membrane involvement including similarities in the development of synovitis, pleural and pericardial effusion, and meningitis. NEUT—neutrophils, LYM—lymphocytes, RF—rheumatoid factor, and C3a and C5a—complement components, * synoviocytes/pleural and pericardial mesothelial/leptomeningeal cells, ** replacement of mesothelial cells with macrophage derived cells. Created with BioRender.com.

Pleural, pericardial, and meningeal membranes can be affected as a part of RA EAMs. It is worth noting that there are some common features related to the inflammation processes in RA, as well as similarities to the synovial inflammatory process (Figure 3). Furthermore, pleural, pericardial effusion, as well as rheumatoid meningitis are linked to high titers of RF, implicating its role in their pathogenesis [125,126,127]. Pleural and pericardial effusions are both frequent manifestations of RA, found in 70% and 30–50% of RA patients respectively. However, symptomatic pleuritis and pericarditis are found in only 3–5% and under 10% of patients, respectively [86,128]. Furthermore, both of these rheumatoid effusions are usually exudative, containing low glucose and complement levels and high protein levels, as well as RF and immunocomplexes [125,129,130]. Cells found in rheumatoid pleural effusions are mostly neutrophils in the first days, which are replaced by lymphocytes 7–11 days later [125]. Tadpole cells and multinucleated giant cells, as well as the replacement of parietal pleural mesothelial cells with a palisade of macrophage-derived cells, are considered pathognomonic and share a resemblance to histological findings in the synovia of RA joints [125]. Both pleural and pericardial tissue were shown to synthesize RF and immunocomplexes (in levels higher than those in the serum) pointing to a local humoral immune response in membranes, rather than the deposition of circulating immunocomplexes [129,131]. Similar cytokine profiles of high TNF-α and low interferon γ, as well as both alternative and classic complement pathways, in addition to the resemblance between immunocomplexes found in rheumatoid pericardial effusion and synovia, suggest similar pathogenesis of local inflammation in synovia and serosal membranes in RA [129,131]. Although we have not found studies of cytokine levels in rheumatoid pericardial effusions, there is a case report of high levels of IL-6 in a rheumatoid pericardial effusion and a case of successful treatment of an effusive–constrictive pericarditis secondary to rheumatoid arthritis with an anti-IL-1 agent (anakinra), suggesting a role for these cytokines, in this extraarticular manifestation, as well as in others [132,133]. Similarly, an elevated effusion/serum IL-6 ratio was found in autoreactive pericarditis, as opposed to viral or neoplastic pericarditis [134]. On the other side, IL-6 has a well-known role in RA joint inflammation [57]. Furthermore, ACPAs share similarities to antibodies against fibronectin and vimentin found in inflammatory myocarditis in animal models, suggesting that, either directly via citrullinated proteins (as already suggested in the previous subheading) or via cross-reactivity to similar proteins, could lead to an inflammatory response of the myocardium and consequently to exudative effusion [77,79,135]. On the other hand, not only inflammation, but hemodynamic effects of lower oncotic pressure as a result of decreased albumin levels in RA patients could also be important in effusion pathogenesis, especially for asymptomatic ones as it was found that hypoalbuminemia was a significant variable linked to asymptomatic rheumatoid pericardial effusion [127]. Rheumatoid meningitis is exceedingly rare, and thus information about its pathogenesis is scarce and mainly dependent on case reports. Pachymeningitis or leptomeningitis, rheumatoid nodules, and vasculitis, including one or rarely all three of these features, are characteristic histopathologic findings in rheumatoid meningitis [126]. Pachymeningitis or leptomeningitis usually consists of nonspecific infiltrates of mononuclear cells, particularly plasma cells. In some cases, multinucleated giant cells are found [126]. These were already mentioned above as typical findings in rheumatoid pleural effusion. As about half of the patients develop rheumatoid meningitis while in a state of low articular disease activity, one could argue that local inflammation plays an important role in rheumatoid meningitis [136]. Cerebrospinal fluid (CSF) analysis does not offer specific findings: usually mild lymphocytic pleocytosis, elevated protein levels, and normal to lower glucose levels are found while findings of elevated RF are inconsistent [126,137]. Markenson et al. suggested in their case report that localization of rheumatoid meningitis at a discrete location with elevated RF and immunocomplex levels found in CSF at this location and not in CSF obtained in a remote region, point to local immune reaction, similar to inflammation found in other rheumatoid effusions [138]. Furthermore, although rheumatoid meningitis may even be the first RA manifestation, it usually manifests in patients with a long history of RA, often with over 10 years of duration and frequently when the effect on joints is already burnt out, adding to the theory of local immune reaction separated from articular or systemic inflammation [126,136]. As for cytokines, Kato et al. found elevated IL-6 in the CSF of a patient with severe rheumatoid meningitis, and its levels correlated with changes in clinical symptoms implying a possible role of IL-6 in rheumatoid meningitis pathogenesis [136]. The involvement of meninges, but not brain parenchyma, in RA is suggested to be a consequence of an autoimmune reaction to collagen [126].These data mainly point to local inflammatory processes in body membranes, sharing similarities with processes in the rheumatoid synovial membrane. However, a low percentage of symptomatic pleuritis, pericarditis, and meningitis and a high share of asymptomatic pleural and pericardial effusions compared to symptomatic synovitis, a hallmark of RA, suggest there could be a mechanism for perpetuating inflammation in synovia compared to other membranes, and that certain triggers could initiate a similar inflammatory response in these membranes.

## 6. Neurologic Manifestations

The involvement of the nervous system in RA includes entrapment neuropathies, ischemic neuropathies caused by rheumatoid vasculitis, and involvement of the cervical spine (atlantoaxial subluxation, basilar invagination), rheumatoid meningitis, and rarely vasculitis or amyloidosis [13]. Neurological symptoms caused by cervical spine involvement are a result of inflammation (similar to that in peripheral joints) of atlantooccipital and atlantoaxial joints leading to pannus formation and juxta-articular bone erosion, which can cause compression of the spinal cord, nerve roots, cranial nerves, medulla, or vertebral arteries (depending on the affected joint) either by subluxations (which is more frequent) or in rare cases directly by inflammation tissue [139]. Similarly, compression neuropathies are caused by the compression of peripheral nerves (most often the median nerve, causing carpal tunnel syndrome), by swelling of adjacent tendons due to tenosynovitis, or by inflamed joint synovia [140]. As opposed to these neurological manifestations of RA which are consequences of joint inflammation in RA, rheumatoid meningitis and ischemic neuropathies are caused by extraarticular inflammation. The pathogeneses of rheumatoid meningitis and serositis in RA seem to bear a certain resemblance, and in order to point out these similarities they are described in the section “Membrane involvement” of this article. Ischemic neuropathies due to rheumatoid vasculitis usually present as mononeuritis, mononeuritis multiplex, multifocal, sensory and motor neuropathy, and distal symmetric sensory neuropathy. They are a consequence of vasculitic changes in the vasa nervorum [139] and the pathogenesis of these changes is described in the section “Vasculitis” of this article. 

## 7. Ocular Involvement

Effects on the eyes are found in about 18% of RA patients [141]. The most common eye evolvement in RA is keratoconjunctivitis sicca (KCS) followed by episcleritis, scleritis, peripheral ulcerative keratitis (PUK), and anterior uveitis [141,142]. Although the pathogenesis of these conditions is not completely understood, studies point to possible inflammatory mechanisms of these conditions in RA. The density of peripheral and central corneal Langerhans cells (LC) is higher in RA patients. In addition, LC in RA patients exhibited increased dendrites. However, in patients treated with systemic glucocorticoids and TNF-α inhibitors, these changes were not observed [143]. However, in a recent study, an increased density of corneal LC was also observed in RA patients, but with a higher density of immature LC. Furthermore, the density of LC correlated with disease activity and with corneal nerve fiber loss indicating a role of inflammation in corneal nerve degeneration [144]. Villani et al. compared levels of cytokines (TNF-α, IL-1, IL-8, and IL-8) in the tear fluid of RA patients with KCS before and after systemic anti-inflammatory treatment. The results showed a decrease in IL-1 and IL-6 in patients with secondary Sjögren’s syndrome (SSII) but not in those with KCS without SSII suggesting that local pathogenic processes, independent of systemic inflammation, could be involved in these patients [145]. Another study showed elevated levels of IL-17 in the tears of RA patients with KCS compared to healthy controls [146]. In murine models of dry eye, IL-17 was linked to the disruption of corneal barrier function through the induction of certain matrix metalloproteinases (MMP-3 and MMP-9) [147]. Episcleritis is hypothesized to be a result of non-granulomatous inflammation of superficial blood vessels of the episcleral tissue resulting in their dilatation and perivascular infiltration [148,149]. Although not offering direct experimental data, disease mediators inducing tissue damage in the eye (e.g., IL-2, IL-6, IL-8, MMP, NO, TNF, and TGF-β) are also found to affect joints in RA patients [150]. Therefore, episcleritis in RA could be explained by the disruption of normal pro- and anti-inflammatory tear cytokine profiles through systemic inflammation [149]. While episcleritis refers to the inflammation of a thin layer of tissue between the conjunctiva and sclera, scleritis is a more serious condition affecting the whole sclera. Nishio et al. in their study on arthritis mice models showed that macrophages, plasma cells, deposition of immune complexes, and growth of blood and lymphatic vessels could play a role in RA scleritis pathogenesis. In addition, treatment targeted at macrophages, such as IL-6 and TNF-α inhibition, and at B-lymphocytes and not to T-lymphocytes, showed better efficacy in murine models and joined case reports of RA patients with scleritis [151]. Furthermore, TNF-α and IL-1, well-known cytokines in RA pathogenesis, have also been found to have a role in the pathogenesis of necrotizing scleritis and PUK, and part of this role could be through increasing MMP production [149]. PUK pathogenesis is also proposed to be linked with the activation of CD4+ cells leading to antibody production and immunocomplex deposition causing complement activation and thus accumulation of neutrophils and macrophages which produce MMPs [149,152]. In both cases, MMPs cause extracellular matrix protein degradation and tissue necrosis [152]. 

## 8. Hematologic Manifestations

Different hematologic manifestations can occur in RA, such as anemia, neutropenia (particularly in Felty syndrome), thrombocytopenia, thrombocytosis, eosinophilia, and hematological malignancies. Lymphadenopathy may also be observed in the active disease, with a pathohistological appearance of benign follicular hyperplasia [3]. Among these, anemia is the most common and is in general one of the most frequent EAMs of RA. Both anemia and thrombocytosis can correlate to disease activity, specifically to joint inflammation. There are multiple pathophysiological mechanisms contributing to different types of anemia in RA [3]. Anemia of chronic disease in RA is thought to result from cytokine-mediated inhibition of iron utilization, apoptosis of erythroid progenitors, and inhibition of erythropoietin production [153]. Specifically, TNFα-mediated apoptosis of erythroid cells has been proposed by some authors [154]. Voulgari et al. showed that anemic RA patients had significantly higher serum levels of TNFα, IL-1β, and IL-6 compared to non-anemics and that these cytokines had inhibitory effects on bone marrow cultures [155]. Iron deficiency anemia can also occur in RA and is usually the result of long-term use of non-steroidal anti-inflammatory drugs (NSAIDs) which leads to gastrointestinal blood loss. Both the anemia of chronic disease and iron deficiency anemia can coexist in RA patients [153]. Functional iron deficiency may also occur in anemia of chronic disease, due to overexpression of hepcidin (the main iron regulatory hormone) which leads to the storage of iron, instead of it circulating, resulting in iron-restricted erythropoiesis [156]. Neutropenia in RA is most frequently discussed as part of the largely RA-specific entity, Felty syndrome, which is caused by a number of different pathophysiologic mechanisms [157]. 

### Felty Syndrome

Felty syndrome (FS) is a rare extra-articular manifestation of seropositive RA characterized by neutropenia and splenomegaly, leading to severe and recurrent infections, especially of the skin and respiratory system. It usually occurs in longstanding diseases, often in patients with other EAMs of RA [5]. There are several mechanisms described leading to the hallmark feature of FS, neutropenia: inhibited granulopoiesis in bone marrow (BM), decreased G-CSF-induced maturation, peripheral neutrophil destruction, and sequestration of neutrophils in the spleen [157]. Studies suggest that suppression of granulopoiesis could be both cell-mediated, as T-cells and monocytes were shown to inhibit granulopoiesis in the BM in FS, and by non-immunoglobulin humoral factors as sera of FS patients caused bone marrow inhibition in mice [158]. Interestingly, BMs in most patients were hypercellular with left-shifted maturation, pointing to increased neutrophil destruction, while in the minority of FS patients, it was hypocellular, pointing to defects in granulopoiesis [157,159]. Furthermore, in the context of FS, T-cell large granular lymphocyte leukemia (T-LGL) also has to be mentioned, as today it is postulated by some that these two are different ends of a spectrum of the same disease [160,161]. It is suggested that neutropenia in T-LGL patients and FS patients with hypocellular bone marrow have the same origin, which is believed to be caused by inhibited granulopoiesis by T-cells [159]. Adding to this theory of different ends of the same spectrum is a study conducted by Savola et al. which found a similar percentage of somatic STAT3 mutations and cytokine profiles, but smaller T-cell clone expansion, in FS patients compared to T-LGL patients [162].

STAT-3 mutation is proposed to lead to the expansion of the monoclonal cytotoxic lymphocyte population and therefore to their cytotoxic activity in BM and the production of proinflammatory cytokines leading to neutropenia [162,163]. Furthermore, impaired G-CSF-induced maturation caused by anti-G-CSF antibodies and lower sensitivity of myeloid cells to G-CSF could also contribute to neutropenia in FS [164]. As mentioned above, another important mechanism of neutropenia in FS is peripheral destruction. Human neutrophils in vitro showed increased activation and endothelial adhesion when exposed to sera of FS patients, and neutrophil count in mice was observed to decrease after FS sera injection as opposed to the reaction to healthy controls’ sera, which caused a neutrophil count rise [165,166,167]. However, this reaction in mice was not observed after immunocomplex removal [167]. Neutrophils in FS patients may be bound to immunocomplexes, which could activate them, cause them to adhere to endothelial cells, and lead to the development of oxidative apoptosis due to the presence of precipitated immunocomplexes [159]. Dwivedi et al. proposed a theory of FS pathogenesis, based on their discovery of antibodies to deaminated histone (a product of neutrophil NETosis) in FS patients. According to this theory, the inflammatory state existing in RA patients and proneness to infection leads to neutrophil activation and NETosis, exposing deaminated histones and triggering autoantibody production, which then may by themselves or by forming immunocomplexes stimulate neutrophils and thus form a vicious circle of neutrophil depletion [168]. Although histological findings and good response to splenectomy in some patients point to the role of neutrophil sequestration in FS, not all patients present with splenomegaly or respond to splenectomy, suggesting only a partial role of the spleen in FS pathogenesis [165,169,170].

## 9. Osteoporosis

While traditionally viewed as a comorbidity of RA, osteoporosis is now understood to involve a complex interplay between immune cells and cells responsible for bone remodeling, as revealed by recent advancements in the understanding of its pathogenesis in RA. RA can also cause bone loss, both locally to the affected joints, juxta-articularly, and systemically [171]. The pathogenesis of systemic and local bone loss in RA is similar. Local bone loss in RA refers to the destruction of bone tissue that occurs in the immediate vicinity of affected joints [172]. This bone loss is a consequence of the inflammatory process that occurs in the synovial tissues, which releases cytokines and other inflammatory molecules that stimulate bone resorption by osteoclasts, the cells responsible for breaking down bone tissue [171,172]. Inflammatory molecules such as TNFα, IL-6, and IL-17 can promote the breakdown of bone tissue and have been found to have higher levels in RA patients who had osteoporosis when compared to those without the condition [173,174]. Conversely, the levels of IL-4 and IL-10 were lower in RA patients with osteoporosis [173]. Qiu et al. also proposed that a combination of factors, including disease activity score (DAS28), along with levels of IL-4, IL-10, and IL-17, could be used to predict the incidence of osteoporosis in individuals with RA [173]. Studies have also shown that RA patients with a Simplified Disease Activity Index (SDAI) score above 3.3 are at a higher risk of fragility fractures compared to those who are in remission [171]. A study conducted on individuals with RA showed that senescent CD4+ cells produced higher levels of receptor activator of nuclear factor kappa-B ligand (RANKL) than CD4 + CD28+ cells. CD4+ T helper 17 (Th17) cells, which are known to play a role in autoimmunity, also trigger the process of osteoclastogenesis [174]. They generate two essential factors, RANKL and IL-17, with the latter stimulating the production of RANKL by fibroblasts and osteoblasts. Additionally, Th17 cells promote the generation of macrophage colony-stimulating factor (M-CSF) and RANKL by osteoblasts and stromal cells, and they also induce the expression of TNFα and RANK in osteoclasts [172]. RANKL is a protein that plays a crucial role in the formation and activation of osteoclasts, which are responsible for the breakdown of bone tissue [172]. This finding highlights a mechanistic link between T-cell senescence and the generation of osteoclasts, which can lead to osteoporosis in RA patients. ACPAs are believed to play a role in mediating bone loss in RA. This is supported by evidence that RA patients often have low bone mass at the onset of the disease. ACPAs can contribute to systemic bone loss by binding to citrullinated vimentin on the surface of osteoclasts, which can promote the differentiation and activation of these bone-resorbing cells [171]. In a study by Kleyer et al., it was found that individuals with ACPA-positive RA had significantly lower bone mineral density (BMD) compared to ACPA-negative individuals [175,176]. Systemic loss of bone density in RA is thought to be due to a combination of factors, including chronic inflammation, the use of glucocorticoid medications, and decreased physical activity. The effect of glucocorticoids on bone health in RA is still a topic of controversy [171]. While some studies have reported a neutral effect of glucocorticoids on BMD in individuals with early and active RA, others have suggested that glucocorticoids may have unfavorable effects on bone health. For instance, a meta-analysis by Wang et al. indicated that glucocorticoids in RA may increase the risk of osteoporosis, particularly at high doses [177]. Studies have shown that the prevalence of osteoporosis is higher in postmenopausal and male RA patients compared to premenopausal patients, and Vitamin D deficiency has also been found to be significantly associated with an increased risk of new clinical and new osteoporotic fractures in women with RA who are over the age of 50 [178,179]. In addition to the general risk factors associated with osteoporosis, such as advanced age and vitamin D deficiency, individuals with RA are also at an increased risk of developing bone fragility due to the presence of sarcopenia. Sarcopenia, which refers to the loss of muscle mass and strength that commonly occurs in RA, can lead to an increased risk of falls and subsequent fractures [180]. 

## 10. Vasculitis

Rheumatoid vasculitis (RV) is one of the rare EAMs of RA that can affect small and medium-sized vessels of various organ systems. It most commonly affects the skin (causing nail fold infarcts, purpura, digital ischemia, livedo reticularis, etc.) and the peripheral nervous system [181]. RV is defined by leukocytoclastic necrosis or cellular infiltrate in the vessel wall. A perivascular infiltrate with more than three cell layers is highly specific for distinguishing RV from RA without RV [182]. RV patients are RF- and ACPA-positive, indicating the autoantibodies’ possible role in RV [183]. RV symptoms occur 10–14 years after RA diagnosis when joint inflammation is low, showing independence of joint involvement [183]. Certain HLA-DRB1 SE genotypes (such as 0401/*0401, *0401/*0404, and *0101/*0401) have been shown to be linked to RV [184]. Smoking is a risk factor for RV, similar to that described in lung involvement. The exact pathophysiological mechanisms are unclear, but smoking may lead to endothelial dysfunction, vascular occlusion, and p53 mutations [183,185]. Circulating RF immunocomplexes bound to C3 and low complement levels in RV patients suggest immunocomplex deposition in the vessel wall and complement activation leading to inflammation and destruction [186,187]. Th1 inflammatory response is present in RV with the expression of TNF-α, ICAM1, and E-selectin [188]. CD4 + CD28null T-cells and the KIR2DS2 gene are also implicated in RV pathogenesis [61,181,189]. 

## 11. Renal Manifestations

Renal manifestations in RA occur rarely, especially with recent advancements in RA treatment, as newer medications have led to better disease control and a lower incidence of renal involvement (such as amyloidosis) in RA patients [190]. However, some previously used disease-modifying antirheumatic drugs (DMARDs) and the high usage of non-steroidal anti-inflammatory drugs (NSAIDs) have contributed to renal disease in RA patients [190]. Around 7–10% of RA patients have renal impairment, but it is not usually directly caused by RA [191]. Daoussis and al. have shown that CKD in RA patients is related to traditional cardiovascular risks such as older age, dyslipidemia, and serum uric acid, rather than disease activity or duration. Additionally, the presence of extra-articular disease (excluding rheumatoid nodules) has also been linked to CKD in RA patients [192]. As for genetic predisposition, an association of serological HLA-DR15 and genotypic HLA-DRB1*1501 with renal involvement in RA patients has been observed and it was linked to different pathohistological types of kidney disease [193]. The most common histological findings of kidney biopsies in RA patients are mesangial glomerulonephritis (GN), membranous nephropathy (MN), and secondary amyloidosis [194]. Korpela et al. found two patterns of mesangial GN in RA patients: granular IgM deposits correlated with IgM RF levels, and granular IgA deposits with C3 complement component correlated with disease severity and duration. Patients with both patterns had higher RF titers than RA controls without mesangial GN [195]. Higher levels of IL-6 in RA patients could also be involved in mesangial GN pathogenesis, as it induced mesangial proliferation in animal models and was elevated in non-RA patients with mesangial GN [190,196]. Membranous nephropathy in RA patients is related to certain DMARDs, such as bucillamine, D-penicillamine, and gold, suggesting it is not directly caused by specific, RA-related mechanisms [197,198,199]. By contrast, secondary amyloidosis is caused by uncontrolled RA activity, leading to high levels of proinflammatory cytokines that cause SAA production by hepatocytes. Serum amyloid A then deposits in kidneys, leading to their dysfunction, especially in genetically predisposed individuals [190]. 

## 12. Conclusions

The clinical presentation of RA is heavily influenced by its EAMs, which have a damaging effect on patient life quality and mortality. To advance the treatment of RA and improve patient outcomes, further research is needed to gain a better understanding of the pathogenesis of the EAMs. The pathophysiological mechanisms of several frequent EAMs are discussed in this review, in order to provide an overview and aid further advancements in research (Table 1). The heterogenicity of the disease, as well as the fact that some of the cited studies used small groups, models, or in vitro experiments, present clear limitations of this comprehensive review (Table 2). It is important to recognize the individual needs of RA patients, which may include multiple comorbidities, and to provide personalized therapeutic options with consideration of their safety. A thorough evaluation of patients and their respective EAMs is essential to provide effective treatment and improve outcomes.

## Figures and Tables

**Table 1:** Overview of RA-associated EAMs and pathogenic mechanisms discussed in this article.

Extraarticular Manifestation	Proposed Pathogenic Mechanisms
Atherosclerosis	Traditional risk factors, immune dysregulation, systemic inflammation, hormone dysregulation, genetic predisposition, and altered endothelial progenitor cell function [25] Endothelial dysfunction and activation: -↓NO caused by ↑TNF-α, ↓ADMA, ↑LOX-1 [27,28,29,30,31,32,33,34] -microparticles linked to ↑adhesive molecules [35] “Lipid paradox”: -↑L5 LDL-C fraction linked to ↑foam cell formation and ↑oxLD [44,45] -↑piHDL leading to ↓HDL cholesterol efflux activity [47,48] -↓HDL cholesterol efflux activity caused by ↑MPO activity [49,50,51] ↑CD14 + CD16+ monocyte level by altered cholesterol efflux in HSPCs [53,54,55] ↑Monocyte recruitment and procoagulant activation by IL-6 activity [56,57,58] ↑CD4 + CD28null T-cell level [64] ↑NETosis induced by RA serum, probably via IL-6, TNF-α [66,67]
Non-ischemic heart disease	↑Inflammation on PET-CT and MR scans [69,70,71] ↑Protein citrullination and ACPA linked to ↑LV mass [77,78,79,80] ↑TNF-α linked to LV dysfunction and remodeling via ↓β-adrenergic responsiveness, ↑MMPs, ↑sphingomyelinase pathway [72,73,74] ↑IL-1 linked to depressed myocardial activity by ↓β-adrenergic responsiveness [75,76]
Interstitial lung disease	Genetic predisposition (HLA-DQB1*03:01, HLA-DRB1*15, and HLA-DRB1*16, SFTPC, RTEL1, and TERT gene mutations) [94,95] Repeated airway epithelial cell damage, mainly by smoking (PAHs linked to ↑IL-6; nicotine linked to ↑NETosis) leading to dendritic cell autoimmunity by abrogation of autoantigen tolerance, autoantibody production, T-cell activation [14,96,101,102,103] ↑IgA ACPAs in sputum and BAL indicating respiratory mucosa as origin of RA-related autoimmunity [91,92,93] Th17 pathway leading to lung ECM genesis and fibrosis [99,100] ↑ACPAs by immunocomplex formation leading to ↑ IL-6, IL-8, TNF, and NETosis [14,97,98] Dysbiosis and GERD leading to introduction of culprit pathogens into the airway [14,105]
Bronchiectasis	Caused by traction due to ILD parenchymal changes or independently [108] Extensive pulmonary inflammation (by autoimmunity or infections) leading to epithelium damage, ↓mucociliary function and ↑bacterial overgrowth leading to perpetuated inflammation (↑IL-17a, ↑IL-1β, and ↑ IL-8) and closing vicious circle [110,112,113] ↑ACPAs by immunocomplex formation leading to ↑ IL-6, IL-8, TNF, NETosis [97,98,114,115]
Rheumatoid nodules	Immune mediated granulomas surrounded by HLADR^+^ [CD64^+^ and C4d^+^) macrophages, lymphocytes and histocytes [117,120,121] Trauma to small blood vessel leading to RF complexes aggregation, macrophage activation and secretion of proinflammatory cytokines and chemokines leading to fibrin deposition and necrosis [116,117,120] Th-1 driven inflammation (↑IL-1, ↑IL-12, ↑IL-18, ↑ IL-15, and ↑IL-10 in pulmonary nodules) similar to RA synovial lesions [124]
Membrane involvement	Rheumatoid pleural, pericardial, meningeal inflammation share mutual features and similarity to rheumatoid joint inflammation [125,129,131] Locally produced RF, immunocomplexes leading to complement activation, ↑Il-6, ↑TNF-α [129,131] Serosal effusion caused by ↓albumin level leading to ↓oncotic pressure [127] ACPA leading to myocardial inflammation and exudation in pericardium [77,79,135]
Neurologic manifestations	Entrapment neuropathies, cervical spine damage caused by joint inflammation and destruction [139,140] Ischemic neuropathies caused by vasculitis of vasa nervorum [139]
Ocular involvement	↑corneal Langerhans cell density linked to corneal nerve degeneration [144] ↑IL 17, TNF-α linked to ↑MMP and ECM degradation [146,147,149] Non-granulomatous inflammation of superficial blood vessels of episcleral and perivascular infiltration [148,149] Scleritis: ↑plasma cells, immunocomplexes, ↑IL-6, ↑TNF-α [151] PUK: CD4+ cell activation leading to immunocomplex deposition, complement activation and ↑MMP leading to ECM degradation [149,152]
Anemia	Cytokine-mediated inhibition of iron utilization, apoptosis of erythroid progenitors, inhibition of erythropoietin production [153] Bone marrow suppression by TNFα, IL-1β, and IL-6 [155] Available iron storage from circulation by hepcidin [156]
Felty syndrome	Inhibited granulopoiesis in BM by somatic STAT-3 mutation induced cytotoxic T-cells [159,162,163] Impaired G-CSF induced neutrophil maturation [164] Peripheral neutrophil destruction—vicious circle of inflammation induced NETosis leading to autoantibodies production to NET products, immunocomplex formation, and perpetuated neutrophil activation [168] Sequestration of neutrophils in spleen [165,169,170]
Osteoporosis	Cytokines TNFα, IL-6, and IL-17 stimulate bone resorption by osteoclasts [171,172,173,174]. Senescent CD4+ cells produce RANKL, Th17 cells trigger osteoclastogenesis [174] ACPAs promote differentiation and activation of osteoclasts [171].
Vasculitis	Genetic predisposition (HLA-DRB1 SE 0401/*0401, *0401/*0404, and *0101/*0401) [184] Smoking—endothelial dysfunction, vascular occlusion, p53 mutations [183,185] ICs deposition, complement activation result in vessel wall inflammation [186,187] Th1 inflammation with TNF-α, ICAM1, and E-selectin expression [188] Implication ofCD4 + CD28null T-cells and KIR2DS2 gene [61], 181,189]
Renal manifestations	Older DMARDs, NSAIDs, traditional CV factors are important risk factors [190,192] Genetic predisposition (HLA-DRB1*1501) [193] Most common PHD: mesangial GN, MN, secondary amyloidosis [194] Mesangial GN: ↑serum RF titers, ↑IL-6 [190,195,196] ↑↑inflammation leading to ↑ SAA and secondary amyloidosis [190]

NO—nitric oxide, TNF-α—tumor necrosis factor α, ADMA—asymmetric dimethylarginine, LOX-1—lectin-like oxidized low-density lipoprotein (LDL) receptor-1, MPO—myeloperoxidase, HSPC—hematopoietic stem and progenitor cells, LDL-C—low-density lipoprotein cholesterol, oxLDL—oxidized low-density lipoproteins, HDL—high-density lipoproteins, IL—interleukin, NETosis—formation of neutrophil extracellular traps, ACPA—anti-citrullinated protein antibody, PET-CT—positron emission tomography–computed tomography, MR—magnetic resonance, LV—left ventricle, BAL—bronchoalveolar lavage, GERD—gastroesophageal reflux disease, ILD—interstitial lung disease, RF—rheumatoid factor, MMP—matrix metalloproteinases, ECM—extracellular matrix, PUK—peripheral ulcerative keratitis, BM—bone marrow, G-CSF—granulocyte colony-stimulating factor, ICs—immunocomplexes, DMARDs—disease-modifying anti-rheumatic drugs, NSAIDs—non-steroidal anti-inflammatory drugs, PHD—pathohistological diagnosis, GN- glomerulonephritis, MN—membranous nephropathy.

**Table 2:** Overview of studies, their type, and point of interest referenced in this review article.

Condition/EAM	Mechanism Studied (Molecular/Cellular/Both)	Type of Study	Number of Patients/Controls	Points of Interest of Studies Used in This Review Article	References
**Atherosclerosis**	Molecular	In vitro, murine, review	N/A	*NO, TNF, ADMA, LOX-1, IL-6*	[26,27,28,30,31,33,34,36,37,38,56]
	Molecular	Systematic review	582 */612 **	*ADMA*	[29]
	Molecular	In vitro/murine/cross-sectional	9 */6 **	*TNF-α, LOX1/NFκB/Arg2*	[32]
	Molecular	Prospective longitudinal	64 */12 **	*L5 LDL-C*	[44]
	Molecular	Cohort prospective	93 */41 **	*L5 LDL-C*	[45]
	Molecular	Cross-sectional	40 */40 **	*HDL, MPO*	[49]
	Molecular	Cross-sectional	178 */223 **	*HDL, Cholesterol efflux*	[50]
	Molecular	Cross-sectional/in vitro	45 **/44 **	*HDL, MPO*	[51]
	Molecular	Review	N/A	*piHDL*	[47]
	Molecular	Cohort/cross sectional	132 *	*piHDL*	[48]
	Molecular	Cross-sectional	82 */110 **	*HDL antibodies*	[52]
	Cellular	Cross-sectional	125 **	*CD14 + CD16+ monocytes*	[53]
	Cellular	Cross sectional	72 *	*CD14 + CD16+ monocytes*	[54]
	Molecular	Murine/cross-sectional	12 */7 **	*Chol. efflux, HSPC monocyte*	[55]
	Molecular	Murine/cross-sectional	513 families **	*Gp 130 receptor*	[58]
	Cellular	Cross-sectional	87 */33 **	*CD4 + CD28null T-cell*	[64]
	Both	Cohort/in vitro	106 */40 **	*NETosis*	[66]
	Molecular	Cohort/in vitro	20*	*NETosis, IL-6*	[67]
**NIHD**	N/A	Cross-sectional	45 */45 **	*MRI, LV morphology*	[70]
	N/A	Cross-sectional/longitudinal	119 */27 **	*PET-CT, FDG uptake*	[71]
	molecular	In vitro, murine, review	N/A	*TNF-α, IL-1, β-adrenergic, ACPA*	[72,73,76,77,80]
	molecular	Double-blind randomized	23 */19 *	*IL-1*	[75]
	molecular	Cross-sectional	150 *	*ACPAs*	[78]
	molecular	Cross-sectional	17 */15 **	*Citrullination*	[79]
**ILD**	N/A	Review	N/A	*ILD patterns, inflammation origin theories*	[14,90,91]
	molecular	Cross-sectional	14 * + 49 ^±^ /21 **	*ACPAs, IgA ACPAs,*	[92]
	both	Observational	24 */84 **	*ACPAs, lymphocytes*	[93]
	molecular	Case-control	101 */1010 **	*HLA, genetic analysis*	[94]
	molecular	Case-control/cross-sectional	610 */773 *	*HLA, genetic analysis*	[95]
	cellular	Review	N/A	*DCs, T-cells*	[96]
	both	In vitro, murine	N/A	*ACPA ICs, nicotine, GERD EMT,*	[97,105,107]
	molecular	Cross-sectional	55 */36 **	*ACPAs, NETosis, cytokines*	[98]
	molecular	In vitro/cross-sectional	4 */6 **	*IL-17A*	[99]
	molecular	Cross-sectional	8 **	*TGF-β1*	[100]
	cellular	Cohort retrospective	31 *	*Smoking, DCs*	[101]
	molecular	Cohort retrospective/murine/in vitro	4 **/4 **	*Smoking, NETosis*	[102]
	cellular	Cross-sectional/In vitro/murine	24 **/8 **	*Smoking, DCs*	[104]
**Bronchiectasis**	N/A	reviews	N/A	*Pathogenesis theories*	[86,108,110,111]
	N/A	Retrospective	40 * + 7 **	*Infections, biologics*	[109]
	molecular	Cohort prospective	165 **/34 **	*IL-17a, IL-1β, IL-8*	[112]
	molecular	Cross-sectional	40 **/20 **	*TNF-α, IIL-8, IL-10*	[113]
	molecular	Cohort prospective	12 * + 42 ^±±^/15 **	*ACPAs*	[114]
	molecular	Cross-sectional	50 */50 * + 79 **	*ACPAs*	[115]
**RN**	both	Reviews	N/A	*Pathogenesis theories*	[116,117]
	cellular	Observational	11 *	*Histology*	[120]
	cellular	Case report	2 *	*Histology, B-cells*	[122]
	cellular	Cross-sectional	13 *	*Vasculitis*	[123]
	molecular	Cross-sectional	10 *	*IL-1, IL-12, IL-18, IL-15, IL-10*	[124]
**Pleural effusion**	both	Case series/literature review	2 * + 28 *	*Cytology, biochem. analysis*	[125]
	both	Case report/literature review	1 *	*Cytology, RF, ICs, complement*	[131]
	both	Literature review	N/A	*Cytology, RF, ICs, complement*	[129]
**Pericardial effusion**	molecular	Case reports	1 *, 1 *, 1 *	*IL-6, IL-1, ACPA*	[132,133,135]
	molecular	Cross-sectional	51 **	*TNF-α, IL-6, TGF-β1, IFN-γ*	[134]
	molecular	Cross-sectional	20 */67 *	*Albumins, RF*	[127]
**Meningitis**	both	Case report/literature review	48 *	*Cytology, RF, biochem. analysis*	[126]
	molecular	Case report/literature review	24 *	IL-6 ***, inflammation site	[136]
	molecular	Case report	1 *	*RF, ICs, inflammation site*	[138]
**Ocular**	cellular	Cross-sectional	52 */24 **	*LCs*	[143]
	cellular	Cross-sectional	50 */35 **	*LCs, nerve fibers*	[144]
	both	Cohort prospective	24 ****	*LCs, IL-1a,-6, -8, TNF-a*	[145]
	molecular	Cross-sectional	142 **/28 **	*IL-17*	[146]
	molecular	Review articles	N/A	*IL-1, TNF-α, MMP*	[149,150,152]
	both	Murine/case report	1 *	*Cytology, IL-6, TNF-α*	[151]
**Anemia**	both	Cohort prospective/in vitro	45 */24 **	*TNF-α, apoptosis*	[154]
		Cross-sectional	105 */127 *	*TNFα, IL-1β, IL-6*	[155]
	N/A	Review	N/A	*Etiology, Iron-deficiency*	[153]
	Molecular	Case-control	34 */17 *	*Hepcidin, iron*	[156]
**Felty syndrome**	both	Reviews	N/A	*BM histology, T-cells, humoral*	[157,158,159,165]
	molecular	Cross-sectional	14 */20 * + 17 **	*STAT-3*	[162]
	molecular	Cross-sectional	15 */16 *	*G-CSF antibodies*	[164]
	cellular	Cross-sectional	20 */24 * + 34 **	*Neutrophils adherence*	[166]
	molecular	Cross-sectional/murine	8 */6 * + 4 **	*FS sera, mice neutrophils*	[167]
	molecular	Cross-sectional	23 */37 * + 10 **	*NETosis*	[168]
	cellular	Case series	3 *	*Spleen histopathology*	[169]
	N/A	Literature review	118 *	*Splenectomy*	[170]
**Osteoporosis**	N/A	Review	N/A	*Risk factors, inflammation*	[171,172]
	molecular	Cross-sectional	62 */54 *	*TNFα, Il-4, IL-6, IL-10, IL-17*	[173]
	both	Cohort prospective/in vitro	107 */113 **	*T-cells, TNF-α, IL-6, IL-15*	[174]
	molecular	Cross-sectional	15 ^±±^/15 **	*ACPAs*	[175]
	molecular	Cohort	238 *	*ACPAs, RF*	[176]
	N/A	Meta-analysis/Systematic review	46,711 */857 **	*Glucocorticoids*	[177]
	molecular	Cohort prospective	2567 *	*Vitamin D*	[178]
	N/A	Observational retrospective	551 *	*Gender, menopause*	[179]
	N/A	Cohort prospective	535 *	*Fall risk*	[180]
**Vasculitis**	molecular	Review	N/A	*RF, ACPAs*	[183]
	cellular	Cross-sectional	12 */14 *	*Histopathology*	[182]
	molecular	Meta-analysis	129 */1439 *	*HLA-DRB1 shared epitope*	[184]
	molecular	Cohort prospective	11 */11 *	*RF ICs, C3, complement*	[186]
	molecular	Cross-sectional	16 */28 * + 9 **	*RF ICs, complement*	[187]
**Renal**	N/A	reviews	N/A	*Histopathology, treatment*	[190,194]
	N/A	Cohort retrospective	128,062 *	*Renal impairment prevalence*	[191]
	N/A	Cross-sectional	400 *	Risk factors	[192]
	molecular	Cross-sectional	41 */134 *	HLA antigens	[193]
	molecular	Cohort retrospective	37 */37 **	Immunofluorescence, RF, ICs	[195]
	N/A	Cohort retrospective	100 *	medication	[197]
	N/A	Cohort retrospective	158 *	medication	[198]
	N/A	Cohort retrospective	53 *	medication	[199]

* RA patients, ** non-RA patients, *** IL-6 measured only in case report, **** 12 RA patients with and 12 without secondary Sjogren syndrome, ^±^ at risk of RA, 26 seropositive and 23 seronegative, ^±±^ non-RA ACPA positive, N/A—not applicable, NO—nitric oxide, TNF—tumor necrosis factor, ADMA—asymmetric dimethylarginine, IL—interleukin, LOX1—lectin-like oxidized low-density lipoprotein (LDL) receptor-1, NFκB—nuclear factor kappa-light-chain-enhancer of activated B cells, Arg2—arginase type 2, LDL-C—low-density lipoprotein cholesterol, HDL—high-density lipoprotein, MPO—myeloperoxidase, piHDL—pro-inflammatory HDL, HSPC—hematopoietic stem and progenitor cells, Chol.—cholesterol, Gp 130—glycoprotein 130, NETosis—formation of neutrophil extracellular traps, ACPA—anti-citrullinated protein antibody, NIHD—non-ischemic heart disease, PET-CT—positron emission tomography–computed tomography, MRI—magnetic resonance imaging, LV—left ventricle, FDG—fluorodeoxyglucose, RF—rheumatoid factor, ICs—immunocomplexes, ILD—interstitial lung disease, DCs—dendritic cells, EMT—epithelial–mesenchymal transition, GERD—gastroesophageal reflux disease, IFN-γ—interferon γ, biochem.—biochemical, LCs—Langerhans cells, BM—bone marrow, G-CSF—granulocyte colony-stimulating factor, FS—Felty syndrome, HLA—human leukocyte antigen, RN—rheumatoid nodules.

## Data Availability

Not applicable.
